# Mechanistic modelling of COVID-19 and the impact of lockdowns on a short-time scale

**DOI:** 10.1371/journal.pone.0258084

**Published:** 2021-10-18

**Authors:** Danish A. Ahmed, Ali R. Ansari, Mudassar Imran, Kamal Dingle, Michael B. Bonsall

**Affiliations:** 1 Center for Applied Mathematics and Bioinformatics, Department of Mathematics and Natural Sciences, Gulf University for Science and Technology, Hawally, Kuwait; 2 Mathematical Ecology Research Group, Department of Zoology, University of Oxford, Oxford, United Kingdom; National Taiwan University, TAIWAN

## Abstract

**Background:**

To mitigate the spread of the COVID-19 coronavirus, some countries have adopted more stringent non-pharmaceutical interventions in contrast to those widely used. In addition to standard practices such as enforcing curfews, social distancing, and closure of non-essential service industries, other non-conventional policies also have been implemented, such as the total lockdown of fragmented regions, which are composed of sparsely and highly populated areas.

**Methods:**

In this paper, we model the movement of a host population using a mechanistic approach based on random walks, which are either diffusive or super-diffusive. Infections are realised through a contact process, whereby a susceptible host is infected if in close spatial proximity of the infectious host with an assigned transmission probability. Our focus is on a short-time scale (∼ 3 days), which is the average time lag time before an infected individual becomes infectious.

**Results:**

We find that the level of infection remains approximately constant with an increase in population diffusion, and also in the case of faster population dispersal (super-diffusion). Moreover, we demonstrate how the efficacy of imposing a lockdown depends heavily on how susceptible and infectious individuals are distributed over space.

**Conclusion:**

Our results indicate that on a short-time scale, the type of movement behaviour does not play an important role in rising infection levels. Also, lock-down restrictions are ineffective if the population distribution is homogeneous. However, in the case of a heterogeneous population, lockdowns are effective if a large proportion of infectious carriers are distributed in sparsely populated sub-regions.

## Introduction

The novel coronavirus SARS-CoV-2 referred to by the World Health Organization (WHO) as COVID-19 (Coronavirus Disease 2019) is believed to have started from an animal source in Wuhan City, Hubei Province, China in December 2019 [1, 2]. Since then, the disease has spread worldwide, making it a global health emergency [3, 4]. On 11^th^ March 2020, the WHO officially classified the COVID-19 outbreak as a pandemic. As of May 30^th^ 2021, COVID-19 (including variants) has infected 170.73 million individuals of which there are 3.55 million deaths have occurred and 152.73 million have recovered, making the total active infected cases approximately 14.45 million [3]. Whilst most symptomatic cases are mild, characterised by a persistent cough and fever, a significant proportion of cases are more serious, where individuals develop pneumonia—leading to acute respiratory failure, which can possibly be fatal. A combination of vastly different non-pharmacological measures have been adopted by national governments to suppress the growth of the epidemic, such as: travel bans, school closures, social distancing, imposed curfews, household quarantine, complete lockdowns, etc [5]. The extent to which of these strategies are most effective, including their timing, is not entirely clear. Although studies have attempted to identify key intervention policies [6, 7], others have highlighted those which are ineffective [8, 9].

COVID-19 is highly contagious and more infectious than initially thought, where improved estimates have shown that during the early stages of the epidemic spread, the number of infected individuals can double every 2.4 days [10]. The virus continues to spread in a similar way to influenza, via respiratory droplets from coughing or sneezing. Therefore, the primary mode of transmission is attained through a ‘contact’ process, i.e., if susceptible individuals are in close spatial proximity of infectious hosts [11]. Once a person is infected, for most people (approx. 81%), no symptoms will show [12]. For others, the time between exposure to the virus (becoming infected) and symptom onset, is on average 5–6 days, but can range from 2–14 days [13, 14]. It is also estimated that a virus carrier will typically only become infectious around 1–3 days before symptoms appear [15]. How long it takes, and to what extent asymptomatic individuals transmit the disease is not completely understood [16, 17]. The preceding suggests that given contact between susceptible and infectious hosts, for an initial period of approximately 3 days, the only, or at least primary means of the virus spreading will be from the initial infected individual to others, without further transmission. This early period will have qualitatively different virus spreading characteristics as compared to the later stages, where newly infected individuals can also spread the disease. Hence it is of interest to study the short-time dynamics of infection levels and of the subtle interplay between the processes involved on this time scale [18, 19]. Moreover, a better understanding of the mechanisms behind disease transmission and the resulting patterns that emerge during this early period is relevant to the current discussion on the origin of COVID-19 [20], and more generally, can contribute towards the design of epidemic prevention polices once the disease has spread to locations where previously it was absent.

Mechanistic movement models provide an alternative modelling approach to conventional epidemiological models (SIR, SEIR), as a means to better understand the dynamics of disease spread [11, 21–23]. One advantage is that the spatial proximity between individuals is explicitly accounted for through individual movement rules, where susceptible individuals come into close contact with infectious hosts, and are possibly infected. Therefore, movement behaviours and the contact patterns that emerge due to these encounters directly relate to disease transmission. In terms of mathematical modelling, Random Walks (RWs) serve as a useful modelling tool for the movement of individuals in a population across space and time [24, 25]. A basic description is given by the Correlated Random Walk (CRW), where the orientations between successive steps are correlated, resulting in a short-term localized directional persistence (referred to as forward persistence) [26–29]. This means that individuals in the short-term are more likely to keep moving in the same direction than to perform abrupt turns. In the absence of forward persistence, the CRW reduces to the Simple Random Walk (SRW), which can be considered as a special case, so that the movement is uncorrelated and completely random [30, 31]. In the case of a population of non-interacting individuals, such movement processes are known to be diffusive, particularly at large spatial scales [32, 33]. In movement ecology, the CRW is supported by empirical evidence from animal movement data, and thus frequently used to model animal movement paths [29, 34–36]. However, in the case of more complicated movement types, such as that observed for humans, the CRW does not provide an adequate description, but can still serve as a null model. To the best of our knowledge, no epidemiological studies have considered host movement as a CRW—even in disease ecology.

Another conceptual tool for modelling movement is the Lévy Walk (LW), where the individual performs short steps forming clusters, with the occasional longer step in between them [37–39]. If the LW is oriented during the clustering phases, the corresponding movement type is referred to as the Correlated Lévy Walk (CLW). In contrast to the CRW, the movement pattern is much faster, and super-diffusive. It is now generally accepted that some animal species perform LWs [40–42], particularly in context-specific scenarios such as foraging, and known to describe an efficient searching strategy where resources are scarce and randomly distributed [43–45]. Alongside this, there is growing empirical evidence that human movements may also exhibit Lévy type characteristics. Such inferences have been reached from studies on the daily movement patterns of humans, traces of bank notes, mobile phone users’ locations and GPS trajectories [46–50]. Therefore, a LW description could be useful to study a wide variety of challenging issues; such as traffic prediction, urban planning, and in the context of our study, epidemic spread [51]. Despite the clear motivation, few studies have focused on epidemics in populations where the host population performs a LW. As an example, it was demonstrated in [52] that a disease outbreak is more likely for similar density populations where individuals perform the LW, instead of the SRW.

In this theoretical study we use a mechanistic description based on RWs to model the movement of susceptible and infectious hosts in 2D space. We focus on the early stage of epidemic development (∼ 3 days), which is the average time lag time before an infected individual becomes infectious, and thus do not account for further transmission. We demonstrate how the type of movement behaviour (diffusive or super-diffusive) has minimal impact on infection levels. In addition, we simulate lock-down scenarios by confining sub-populations of different densities. Thus, we reveal whether imposing a lockdown is effective in mitigating disease spread, and how this may depend on the spatial distribution of the population.

## Movement models

### Random walk framework

The movement of a walker in 2D space along a curvilinear path in continuous space-time, **x** = **x**(*t*) = (*x*(*t*), *y*(*t*)) can be modelled using a discrete time random walk (RW) which links individual location **x**_*i*−1_ at time *t*_*i*−1_ to the next location **x**_*i*_ at time *t*_*i*_. Discrete time analyses of human telemetry data often work with regular time steps, and therefore we assume that each location is recorded at times *t*_*i*_ = *i*Δ*t*, where Δ*t* is considered as a constant time step, independent of *i*. The step length defined as the distance between any two successive steps is *l*_*i*_ = |**x**_*i*_ − **x**_*i*−1_| with average velocity ${\text{v}}_{i}=\frac{{\text{x}}_{i}-{\text{x}}_{i-1}}{\Delta t}$ and speed $v_{i}=|{\text{v}}_{i}|=\frac{l_{i}}{\Delta t}$ [29, 53]. For an *n*-step RW, the complete movement path which begins at location **x**_0_ can then be expressed through the equation:

$$\begin{array}{c} x_{i}=x_{i-1}+{\left(\Delta x\right)}_{i},\;i=1,2,3,...,n \end{array}$$ (1)

where (Δ**x**)_*i*_ = (Δ*x*_*i*_, Δ*y*_*i*_) is a step vector whose components are random variables for the *i*^*th*^ step along the walk. The individual executes a path of length L=nE[l] with total duration *T* = *n*Δ*t*, and mean speed

$$\begin{array}{c} E\left[v\right]=\frac{L}{T}=\frac{E[l]}{\Delta t}. \end{array}$$ (2)

Any 2D RW can be described in polar co-ordinates, by expressing the components of the step vector (Δ**x**) in terms of step lengths *l* and step orientations (or headings) *θ*, using the transformation:

$$\begin{array}{c} \Delta x=l\text{cos}(\theta ),\;\Delta y=l\text{sin}(\theta ),\;l\in [0,\infty ),\;\theta \in (-\pi ,\pi ] \end{array}$$ (3)

with inverse transformation:

$$\begin{array}{c} l=\sqrt{{\left(\Delta x\right)}^{2}+{\left(\Delta y\right)}^{2}},\;\theta ={\text{atan}}_{2}(\Delta y,\Delta x), \end{array}$$ (4)

where atan_2_(Δ*y*, Δ*x*) is equal to $\text{arctan}\left(\frac{\Delta y}{\Delta x}\right)$ for Δ*x* > 0 and to $\text{arctan}\left(\frac{\Delta y}{\Delta x}\right)\pm \pi$ for Δ*x* < 0. The turning angle *α*_*i*_ can then be measured as the difference between the orientations of two successive steps:

$$\begin{array}{c} \alpha_{i}=\theta_{i}-\theta_{i-1}. \end{array}$$ (5)

On assuming that step lengths and step orientations are neither autocorrelated nor cross-correlated [54], the individual movement can be simulated once the distributions of step lengths λ(*l*) and turning angles *ψ*(*α*) are prescribed.

The mean cosine *c* and the mean sine *s*, defined as:

$$\begin{array}{c} c=E\left[\text{cos}\alpha \right]=\int_{-\pi }^{\pi }\text{cos}\left(\alpha \right)\psi \left(\alpha \right)d\alpha ,\;s=E\left[\text{sin}\alpha \right]=\int_{-\pi }^{\pi }\text{sin}\left(\alpha \right)\psi \left(\alpha \right)d\alpha , \end{array}$$ (6)

both lie between 0 and 1, and are useful statistical parameters that characterize the turning angle distribution *ψ*(*α*). A null mean sine *s* = 0 corresponds to a balanced RW (i.e., left and right turns are equiprobable), in which case *ψ*(*α*) is centrally symmetric. The mean cosine *c* represents the correlation between the orientations of successive steps. A null mean cosine *c* = 0 corresponds to completely random movement (known as a simple RW), and at the other extreme end, *c* = 1 corresponds to straight line (or ballistic) movement [55].

### Simple random walk

The earliest RW models of individual movement are uncorrelated and isotropic (unbiased), referred to as simple random walks (SRW). As a result, the direction of movement is independent of previous directions moved and completely random [24, 30, 31]. In this case, the distribution of turning angles *ψ*(*α*) is uniform and defined over the interval from −*π* to *π* with null mean sine and cosine, i.e., a balanced uncorrelated RW. In general, the SRW provides an oversimplified description for movement, but usually serves as a theoretical baseline model for more complicated movement behaviours [56, 57].

For our modelling purposes, we consider zero-centered Gaussian step increments, so that the distribution for the components of the step vector Δ**x** is:

$$\begin{array}{c} \phi \left(\Delta x\right)=\frac{1}{\sigma \sqrt{2\pi }}\text{exp}(-\frac{{\left(\Delta x\right)}^{2}}{2\sigma^{2}}),\;\phi \left(\Delta y\right)=\frac{1}{\sigma \sqrt{2\pi }}\text{exp}(-\frac{{\left(\Delta y\right)}^{2}}{2\sigma^{2}}),\;\Delta x,\Delta y\in R \end{array}$$ (7)

with mean E[Δx]=0 and variance Var[Δ*x*] = *σ*^2^, and exact same expressions for Δ*y*. Due to isotropicity, the variances are equal and depend on a single parameter *σ* which represents the mobility of the individual [58]. The corresponding RW is a discrete time model of Brownian motion, and has been particularly useful in modelling the movement of insects [53, 58, 59]. Also, note that in the more general case of non-Gaussian increments, the basic requirements are that the distribution of each increment is symmetrical and zero-centered with finite variance. This is to avoid any resulting global biases in the movement path (e.g., Biased RW [24]) or the case of heavy tails (Lévy walks or flights [60, 61]).

It can readily be shown that the corresponding probability distribution functions for step lengths and turning angles (*l*, *α*) are given by:

$$\begin{array}{c} \lambda \left(l\right)=\frac{l}{\sigma^{2}}\text{exp}(-\frac{l^{2}}{2\sigma^{2}}),\;\psi \left(\alpha \right)=\frac{1}{2\pi }, \end{array}$$ (8)

where λ(*l*) is the Rayleigh distribution with mean step length and mean squared step length:

$$\begin{array}{c} E\left[l\right]=\frac{\sigma \sqrt{2\pi }}{2},\;E\left[l^{2}\right]=2\sigma^{2} \end{array}$$ (9)

and *ψ*(*α*) is the uniform distribution ranging from −*π* to *π*, see [53] for a derivation of the equations in (8) from the Gaussian step increments in (7). Note that, a finite variance is always ensured if the step length distribution λ(*l*) decays sufficiently fast at large *l*, and this holds for the Rayleigh distribution whose end tail decays faster than exponential.

### Correlated random walk

If an individual is more likely to persist in the same direction in the short term rather than to perform abrupt turns—then the orientations of successive steps are correlated. As a result, there is a short-term localized directional bias in the movement path (forward persistence), and the corresponding movement process is anisotropic and known as the correlated random walk (CRW) [26–29]. In this case, the turning angle distribution is zero-centered and centrally symmetric with null mean sine if we consider a balanced CRW, and peaked about the mean value. An example of such is the von Mises distribution:

$$\begin{array}{c} \psi \left(\alpha \right)=\frac{e^{\kappa \text{cos}\alpha }}{2\pi I_{0}\left(\kappa \right)},\;I_{0}\left(\kappa \right)=\frac{1}{2\pi }\int_{-\pi }^{\pi }e^{\kappa \text{cos}\alpha }d\alpha \end{array}$$ (10)

which ranges from −*π* to *π*, and the concentration parameter *κ* ∈ [0, ∞) measures the strength of forward persistence [62]. Here, *I*_0_(*κ*) denotes the zero^th^ order modified Bessel function of the first kind, defined in Eq (10). Note that other types of peaked circular distributions which are commonly used include the wrapped or truncated normal or the wrapped Cauchy distribution [63, 64]. For the von Mises distribution, the mean cosine *c* can be computed from Eq (6), so that one gets:

$$\begin{array}{c} c=\frac{I_{1}\left(\kappa \right)}{I_{0}\left(\kappa \right)}. \end{array}$$ (11)

The Mean Squared Displacement (MSD), which is defined as the expected value of the squared beeline distance between an individuals’ initial and final positions in an *n* step RW, serves as a useful metric to analyse movement patterns. For a balanced CRW, this can be computed as:

$$\begin{array}{c} E[R_{n}^{2}]=nE[l^{2}]+2E{\left[l\right]}^{2}\frac{c}{1-c}(n-\frac{1-c^{n}}{1-c}), \end{array}$$ (12)

which is expressed in terms of moments of step length *l*, mean cosine *c* and the number of steps *n*, see [29] for a derivation. In the special case of a SRW (*c* = 0), the MSD reduces to:

$$\begin{array}{c} E[R_{n}^{2}]=nE[l^{2}]. \end{array}$$ (13)

For a large number of steps *n*, one gets:

$$\begin{array}{c} E[R_{n}^{2}]=n(E[l^{2}]+2E{\left[l\right]}^{2}\frac{c}{1-c}), \end{array}$$ (14)

and therefore the CRW is diffusive in the long term as the MSD grows linearly with the number of steps *n* i.e., $E\left[R_{n}^{2}\right]∼n$, and can be related to the diffusion coefficient *D* [24, 65–68] as follows:

$$\begin{array}{c} E[R_{n}^{2}]=4Dn\Delta t. \end{array}$$ (15)

The MSD exists and is finite if the decay in the end-tail of the step length distribution is exponential or faster (referred to as a ‘thin’ tail), and the CRW with Rayleigh distributed step lengths (Eq 8) is an example of such.

**Fig 1:**
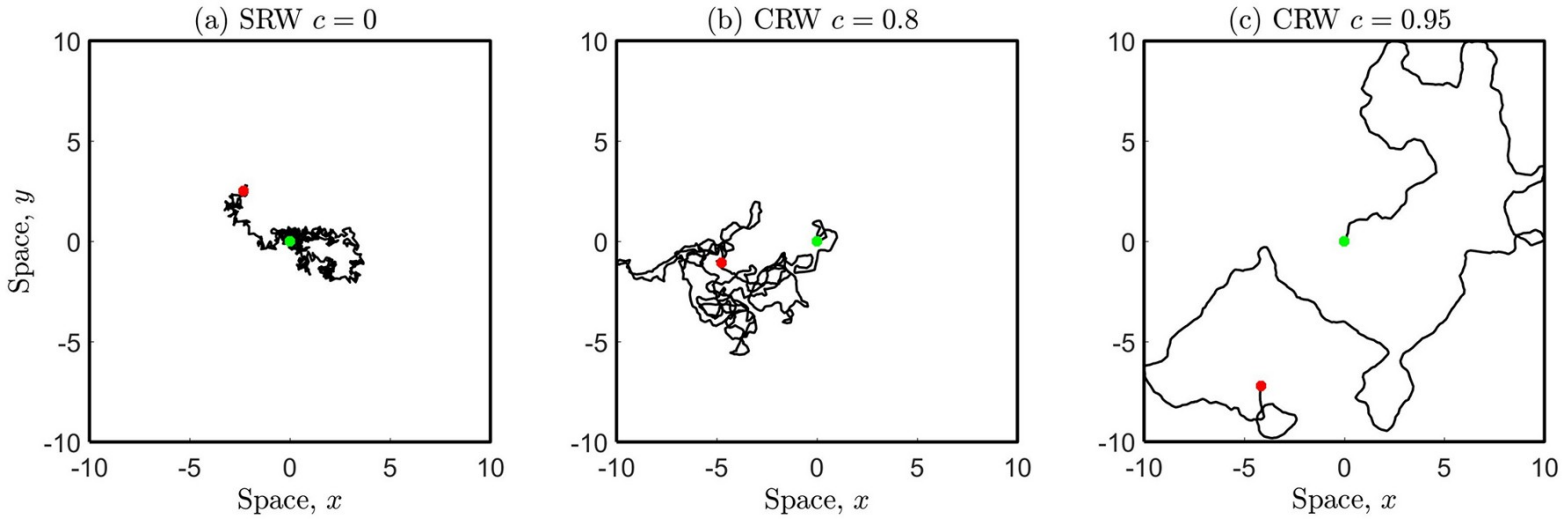
Individual movement paths for the CRW with mobility parameter *σ* = 0.1. (a) SRW *c* = 0 (special case) and (b) CRW *c* = 0.8, (c) CRW *c* = 0.95. Each individual has starting location at (0, 0) (green marker) and executes *n* = 1000 steps, corresponding to time *t* = 25 using a time increment of Δ*t* = 0.025.

Fig 1 illustrates individual movement paths for the SRW and the CRW in confined space. With increasing *c* (or equivalently *κ*) there is an increase in forward persistence, and the path is more diffusive in the long term.

### Lévy walk

Lévy walks (LWs) are a conceptual tool that are often used to model individual human movement paths [46–50]. The main difference between this class of walks, and those prior, is that the asymptotic decay in the end tail of the step length distribution is much more slower (known as a fat or heavy tail), according to the power law:

$$\begin{array}{c} \lambda \left(l\right)∼l^{-\mu },\;1<\mu <3, \end{array}$$ (16)

where *μ* is the Lévy exponent. As a result the MSD is infinite and the corresponding movement process is scale-free. It follows that an individual can execute rare but longer steps composed of clusters of multiple short steps with longer steps in between them [39]. The LW is also known to be super-diffusive, since the MSD increases at a faster rate than in the case of standard diffusion i.e., $E\left[R_{n}^{2}\right]∼n^{m}$ with 1 < *m* < 2. Similar to the SRW, the orientations of successive steps for the LW are uncorrelated and unbiased. One can simply extend this to a correlated Lévy walk (CLW) inclusive of forward persistence during the clustering phases by considering a peaked circular distribution of turning angles, as previously seen for the CRW.

Without loss of generality, we choose to rely on the folded-Cauchy distribution for step lengths:

$$\begin{array}{c} \lambda \left(l\right)=\frac{2\gamma }{\pi (\gamma^{2}+l^{2})}, \end{array}$$ (17)

whose end tail decays quadratically according to a power law $\lambda (l)∼\frac{1}{l^{2}}$ (*μ* = 2). For a CLW, we also consider the von-Mises distribution of turning angles (Eq 10) with mean cosine *c* (Eq 11), where the LW is a special case for *c* = 0. Different movement types modelled by RWs are usually related through their MSDs, but this requires that their MSDs exist and are finite. Two isotropic RWs where at least one of them has an infinite MSD can be compared by relating their distributions of step lengths. More specifically, a condition of equivalence can be sought by ensuring the same characteristic scale length *L* and survival probability ℙ(l>L)=p, (i.e., the probability of occurrence of move lengths longer than *L*). However, this condition can be satisfied for an arbitrary value of *p*. To determine *p* uniquely, an additional constraint is required that minimizes the $L_{2}$ norm, and therefore the distribution movement parameters can be related. Applying this method to compare a SRW with Rayleigh distributed step lengths (Eq 8) and a LW with folded-Cauchy distributed step lengths (Eq 17), one gets:

$$\begin{array}{c} \frac{\gamma }{\sigma }=1.536. \end{array}$$ (18)

This result also applies to compare a CRW to a CLW given that both RWs have the same distribution of turning angles. See Supporting information for more details.

**Fig 2:**
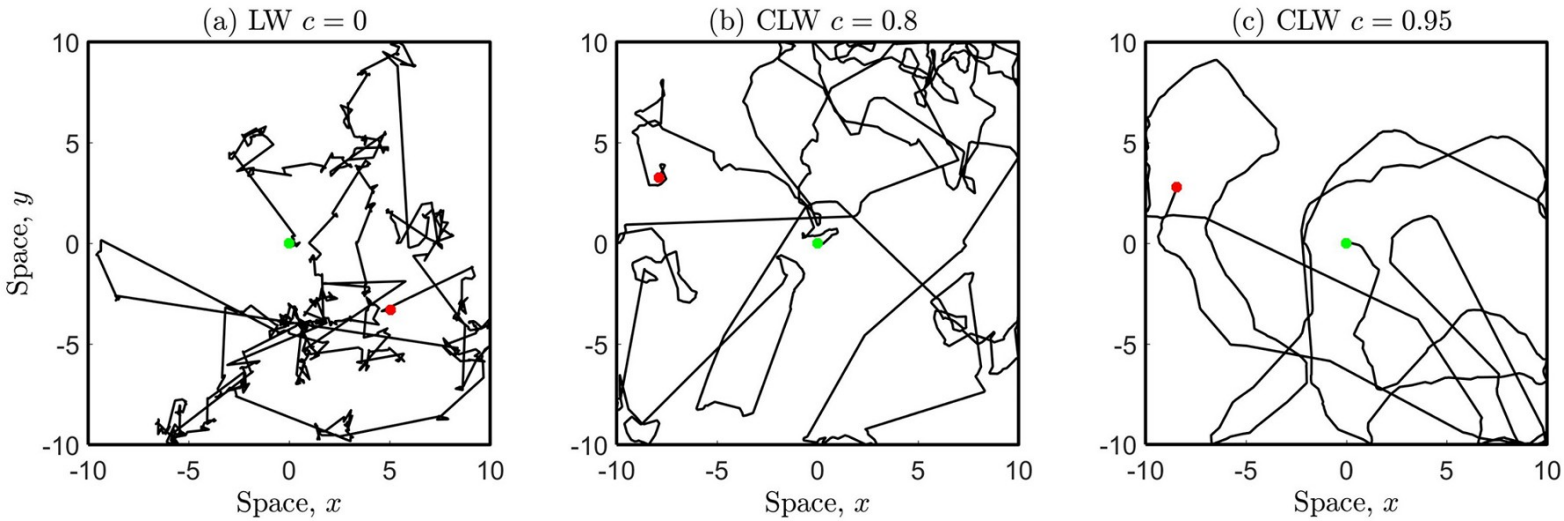
Individual movement paths for the CLW with mobility parameter *γ* = 0.1536. (a) LW *c* = 0 (special case) and (b) CLW *c* = 0.8, (c) CLW *c* = 0.95. Each individual has starting location at (0, 0) (green marker) and executes *n* = 1000 steps, corresponding to time *t* = 25 using a time increment of Δ*t* = 0.025. Note that these RWs are ‘comparable’ to those in Fig 1 since the movement scale parameters are related through the equivalence relation in Eq (18).

Fig 2 illustrates the movement of an individual performing either the LW or the CLW in confined space. The CLW allows for forward persistence during the short-distance clustering phases, with increasing persistency for larger *c*.

## Simulations

### Modelling infection counts

Consider a population of *N* individuals initially homogeneously distributed across a region Ω represented by a square domain of side lengths 2*d*:

$$\begin{array}{c} \Omega =\{(x,y):|x|<d,\;|y|<d\}. \end{array}$$ (19)

How the population disperses in space can be actualised by modelling the movement of each individual independently using an *n* step RW given by Eq (1). The location of the *j*^th^ individual after the *i*^th^ step is thus given by:

$${\text{x}}_{i,j}={\text{x}}_{i-1,j}+{\left(\Delta \text{x}\right)}_{i,j},\;i=1,2,3,\ldots ,n,\;j=1,2,3,\ldots ,N.$$ (20)

Different movement behaviours can be simulated using the movement rules that generate the different types of RWs shown in Figs 1 and 2. During the course of the movement, individuals may encounter the spatial boundary. We consider the boundary to be impenetrable, so that no individuals can leave or enter, and therefore the population is confined at all times. In our simulations, we choose to rely on a ‘no-go’ condition, so that if any individual attempts to overstep the boundary at any instant in time, then an alternative step is chosen at the previous location with completely random orientation [69].

At time *t* = 0, we consider a single individual from amongst the population to be infectious with random initial location ${\text{x}}_{0}^{*}=(x_{0}^{*},y_{0}^{*})$, where each component is drawn from the uniform distribution ranging from −*d* to *d*. The infectious host also performs a RW in 2D space with location at each step determined by:

$$\begin{array}{c} x_{i}^{*}=x_{i-1}^{*}+\Delta x_{i}^{*},\;i=1,2,3,...,n \end{array}$$ (21)

where ‘*’ is included here to distinguish between the infectious host and the remaining susceptible individuals. The movement type is determined by the properties of the random step vector $\Delta {\text{x}}_{i}^{*}$. Since each individual in the population moves independently of each other, including the infectious host, it follows that ${\text{x}}_{i}^{*}$ and **x**_*i*_ are uncorrelated. At each subsequent step, the virus is transmitted to those susceptible individual(s) that are within a close spatial proximity of less than a distance *r* from the infectious host [22] with transmission probability *ϵ*. Clearly, not all individuals that interact with the infectious host are instantaneously infected, host-host contacts can lead to unsuccessful transmission events, referred to as ‘near misses’. More formally, individual(s) are deemed to be infected if:

$$\begin{array}{c} |x_{i,j}-x_{i}^{*}|<r\;\text{and}\;u_{i}<\epsilon \;i=1,2,3,\ldots ,n,\;j=1,2,3,\ldots ,N-1 \end{array}$$ (22)

where *u*_*i*_ is drawn from the uniform distribution ranging from 0 to 1. Given that our focus is on the short-term dynamics, and that there is an estimated time lag of approximately 3 days before an infected individual becomes infectious, those infected individuals are instantaneously removed from the population to ensure that no further interactions are allowed, and the model does not account for infections at future generations. However, the primary infectious host continues to browse throughout the remaining susceptible population as per the RW model, and continues to infect others as a result of further contacts. Infection counts can then be computed as the proportion of total individuals that are infected over this short duration of time.

### Parameter selection for a realistic scenario

Our study aims to provide a theoretical assessment of infection counts, and therefore the results that follow are generally applicable. However, for any interpretations to make sense, model parameters must be chosen that closely reflect a realistic scenario. To obtain a crude estimate for the population density $\rho =\frac{N}{(2d{)}^{2}}$, we set *ρ* equal to 5,700 individuals/km^2^, which is a typical density of a large city, e.g., London (UK) [70]. However, note that the population of the UK is spread unevenly (heterogeneous), and in rural areas, *ρ* can be as small as 50 individuals/km^2^. Also, we consider the population to be distributed over a region of size 1km by 1km (*d* = 0.5). The WHO recommend that individuals must maintain at least a 1m distance between each other, but other organizations such as the CDC have suggested 1.83m (6ft or two arms length), and therefore we consider an interaction radius with value *r* = 1 or 2m [71]. The virus is transmitted to susceptible individuals if they are in close contact with the infectious host with probability *ϵ*. For COVID-19 the probability of infection per exposure is around 0.1 [72, 73], and comparatively, the new COVID variant is estimated to be approximately 56% more transmissible (95% credible interval 50–74%) [74], and therefore we consider both cases with transmission probabilities set to *ϵ* = 0.1, 0.156.

The mobility parameter *σ* can be computed based on an estimated value of the mean speed of the walker. Human walking speeds can be influenced by many factors (e.g., fitness, energetic considerations, time to reach a destination, age etc. [75, 76]), however, the average walking speed of healthy individuals is estimated to vary between 1.2 to 1.4m/s [77, 78], which converts to 4.32 to 5.04km/hr, and therefore we have that:

$$\begin{array}{c} 4.32<E(v)<5.04. \end{array}$$ (23)

If step lengths are modelled to be Rayleigh distributed, from Eqs (2) and (9) we can deduce:

$$\begin{array}{c} \frac{8.64\Delta t}{\sqrt{2\pi }}<\sigma <\frac{10.08\Delta t}{\sqrt{2\pi }}. \end{array}$$ (24)

If we set Δ*t* = 0.025hr, we find that a suitable range for the mobility parameter is 0.0862 < *σ* < 0.1005, and therefore in all further simulations we fix *σ* = 0.1km, i.e., each individual moves an average distance of approximately 0.125km between any two successive locations every 1.5mins, corresponding to an average speed of 5km/hr. On a short-time scale (∼ 3 days), the total duration is *T* = 72hrs, corresponding to *n* = 2880 steps in the walk.

**Fig 3:**
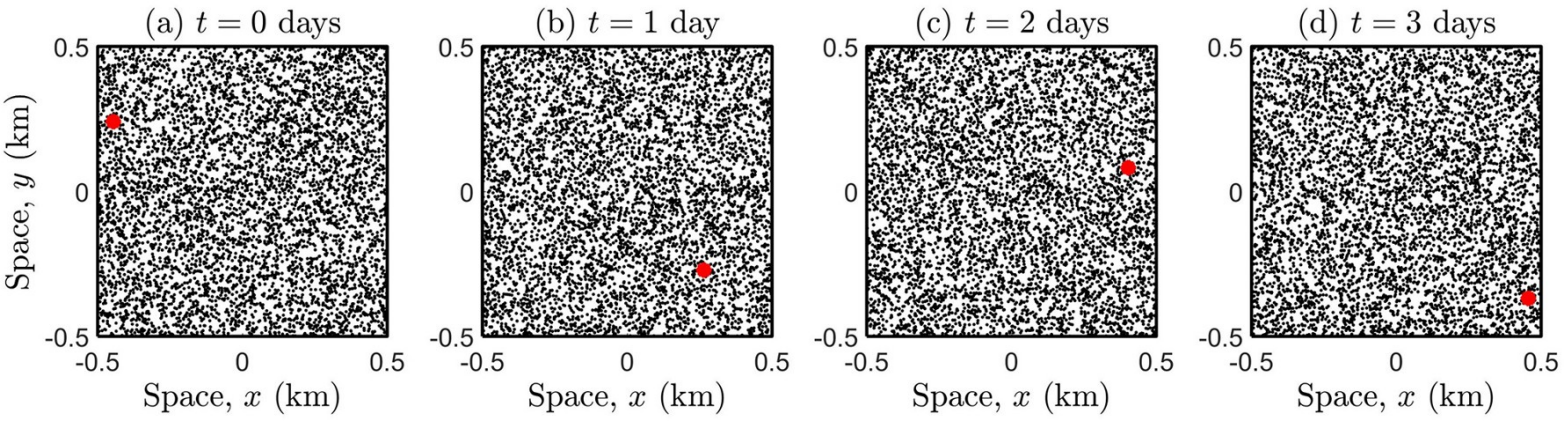
Snapshots of the interplay between an infectious host (red marker) and the susceptible population (with density *ρ* = 5700) at times *t* = 0, 1, 2, 3 days. The initial location of the infectious host is completely random, over a region of size 1km by 1km. Each individual in the population (including the infectious host) performs a SRW with mobility parameter *σ* = 0.1 and null mean cosine. Time is computed as *t* = *n*Δ*t* with Δ*t* = 0.025, so that each individual executes a maximum of *n* = 2880 steps corresponding to *t* = 3 days. Red marker is enlarged for illustrative purposes.

Fig 3 illustrates the simulation methodology. As the infectious host browses through space (red marker), the virus is transmitted to those susceptible individuals (black dots) given a contact (*r* < 1m) with transmission probability *ϵ* = 0.1, and therefore infection counts increase over time (i.e., the total number of infected individuals expressed as a percentage of the total population): (a) 0 (b) 0.0526 (c) 0.0877 (d) 0.1404, obtained from a single simulation run.

### Enforcing lock-downs

Lockdowns have been imposed in many countries and territories to restrict movement, and it is now generally accepted that they are effective at reducing the spread of COVID-19 [79–81]. However, it is not entirely clear how the efficacy of a lockdown may depend on the movement behaviour and the distribution of the population in space, even over a short-time scale in newly infected areas. Here we present different lockdown scenarios for a theoretical assessment of infection counts.

**Fig 4:**
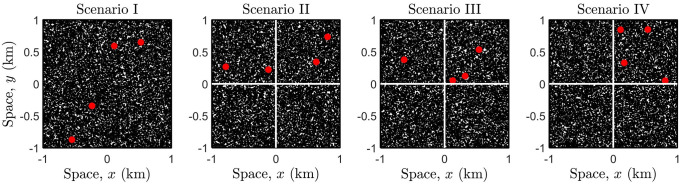
Lockdown scenarios in the case of a homogeneous population spatial distribution.

Fig 4 scenario I shows a population (including infected individuals) with density *ρ* = 5700 individuals/km^2^ which is homogeneously distributed and moves freely according to a RW across the whole region of spatial dimensions 2km by 2km. Four of these individuals are considered to be infectious. In scenarios II-IV, the whole region is partitioned into four adjacent sub-regions of dimensions 1km by 1km (demarcated by the solid lines), illustrating a lock-down by restricting the movement of each sub-population (with equal density) to each of these sub-regions. As a result, potential contacts and hence the transmission of the virus is also confined, and infection counts are computed using the simulation methodology previously described, i.e., through the contact process with assigned transmission probability. The location of each infected individual is uniformly distributed over each respective sub-region, and the number of infectious individuals are alternated to depict different lockdown scenarios.

**Fig 5:**
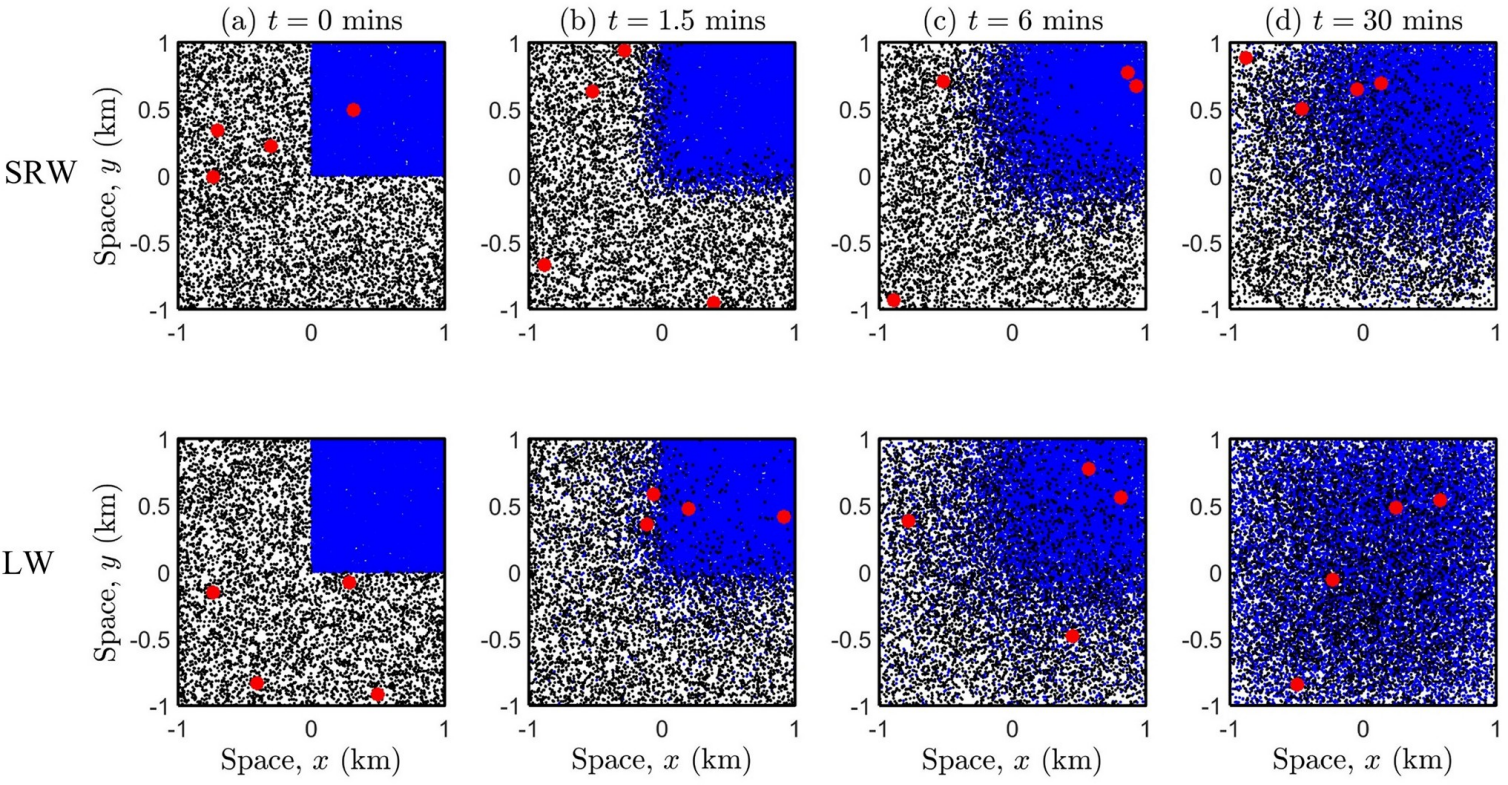
Population mixing due to different movement types in a heterogeneous spatially distributed population, in the absence of a lockdown. The mobility parameter for the SRW is *σ* = 0.1 and for the LW *γ* = 0.1536, see also Eq (18). All other parameter values are the same as that in the caption of Fig 3.

Fig 5 illustrates how a heterogeneous population mixes when no lockdown is imposed, by considering different movement types (SRW or LW). The same set-up seen in Fig 4 scenario I is shown, where the population density for the whole region is *ρ* = 5700, however, the density of the sub-region (top-right) is now ten times larger than the remaining—representing an overcrowded sub-population. Note that due to the super-diffusive properties of the LW, the population mixes much faster, and even after a very short-time (∼30 mins), the population is ‘well-mixed’, cf. plots (d) for SRW and LW.

**Fig 6:**
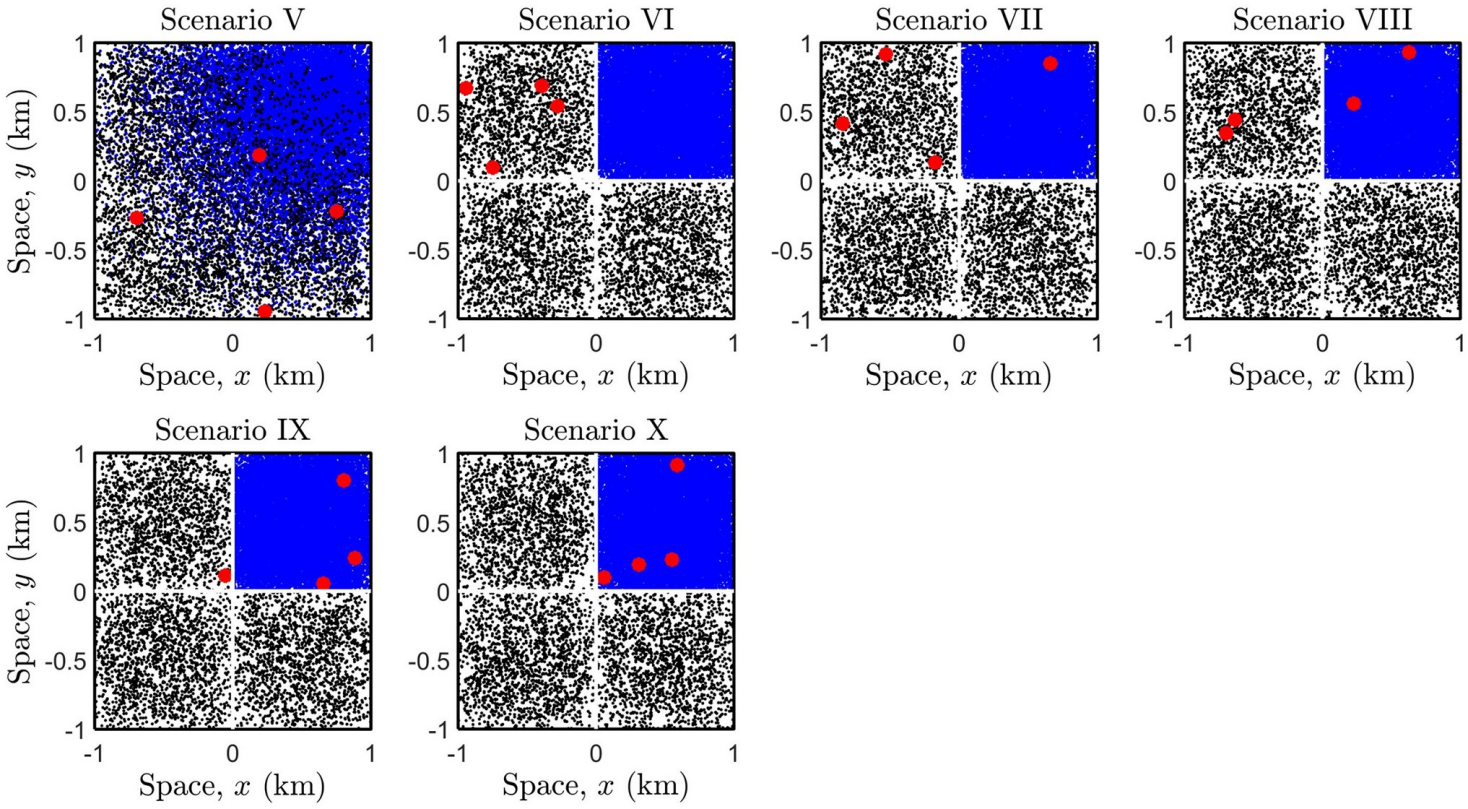
Lockdown scenarios in the case of a heterogeneous population spatial distribution.

Fig 6 illustrates the exact same lockdown scenarios as previously seen in Fig 4, however, the population is now heterogeneous, i.e., the density of the (top-right) sub-region is ten-fold of the remaining. Note that the snapshots are taken at time *t* = 30mins, and scenario V illustrates the free movement of a population where individuals perform a SRW, see also Fig 5 plot (d). In scenarios VI-X, a lockdown is imposed at time *t* = 0, and therefore each sub-population (including infectious individuals) are confined to each respective sub-region at all times.

## Results

### Variation in infection counts due to movement

**Fig 7:**
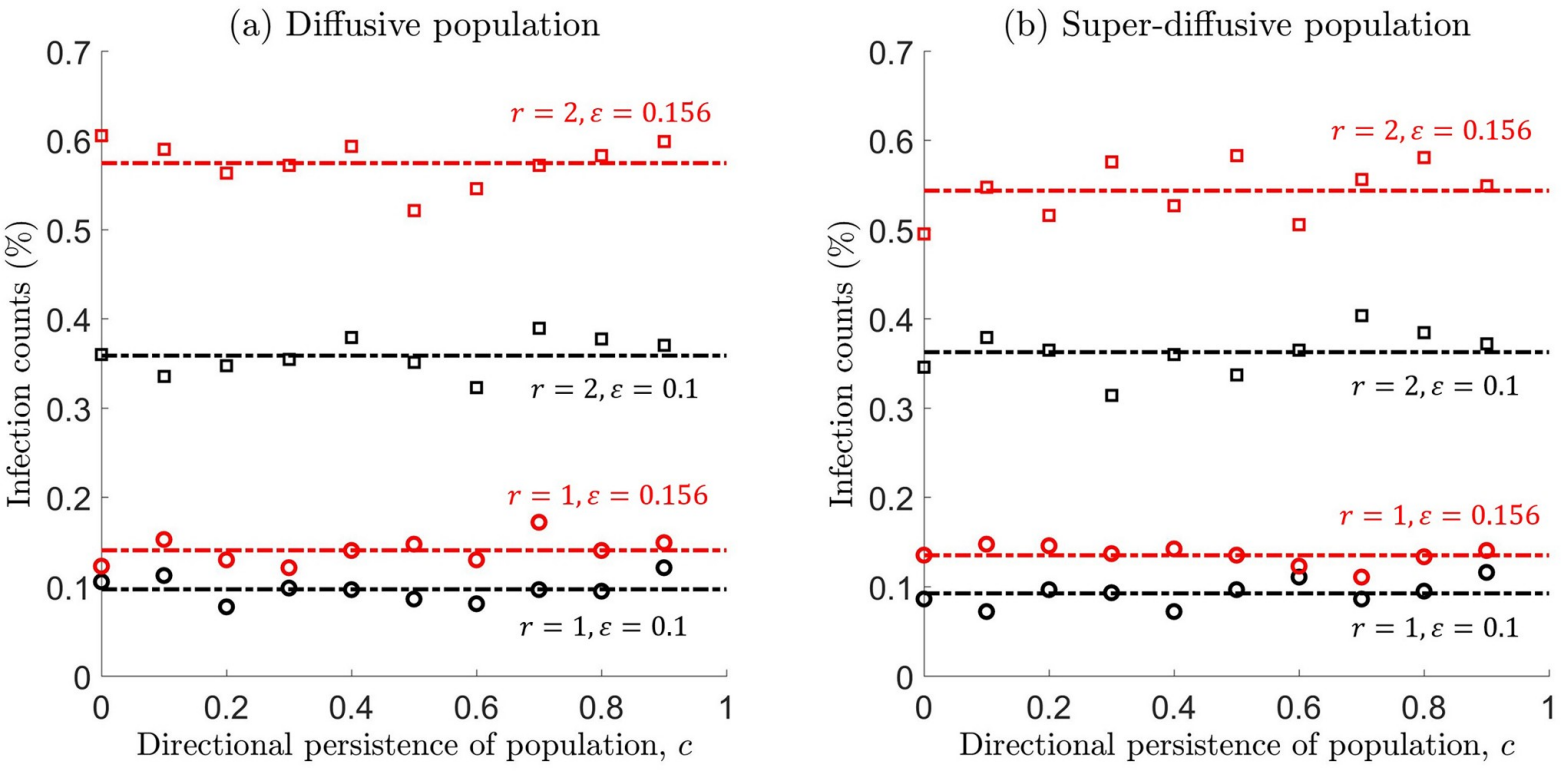
Cumulative infection counts (%) as a function of the mean cosine *c*, whilst considering different modes of population dispersal: (a) diffusive (CRW with *σ* = 0.1) or (b) super-diffusive (CLW with *γ* = 0.1536). These mobility parameters are related through Eq (18), so that infection counts can be compared across different population movement types. Infection counts are recorded at time *t* = 3 days (*n* = 2880 steps in the walk), and computed for different interaction radii *r* = 1, 2m and different transmission probabilities *ϵ* = 0.1, 0.156. All other simulation parameters are the same as that in the caption of Fig 3. Simulations were averaged over ten runs, to reduce the effect of stochastic noise.

Fig 7(a) shows that, on a short-time scale, cumulative infection counts remain approximately constant with an increase in forward persistence, and from Fig 7(b) also remains constant in the case of faster population dispersal. This implies that the movement pattern has no effect on infection counts, and counter-intuitively, the rapid movement of individuals do not explain rising levels of infection. Moreover, infection levels depend on other factors, such as the number of contacts over a fixed time period, size of the interaction radius, and virus transmissibility.

### Impact of lockdowns on a short-time scale

To determine whether lockdowns are effective on a short-time scale, we compare cumulative infection counts across the different scenarios depicted in Figs 4 and 6. We also analyse the impact of considering different interaction radii *r* = 1, 2m, and transmission probabilities *ϵ* = 0.1, 0.156. In addition, we only consider a population that performs a SRW, as it was previously demonstrated that other modes of movement (i.e., inclusion of forward persistence or faster dispersal) has no effect on how infection counts accumulate in the short term, see Fig 7.

**Fig 8:**
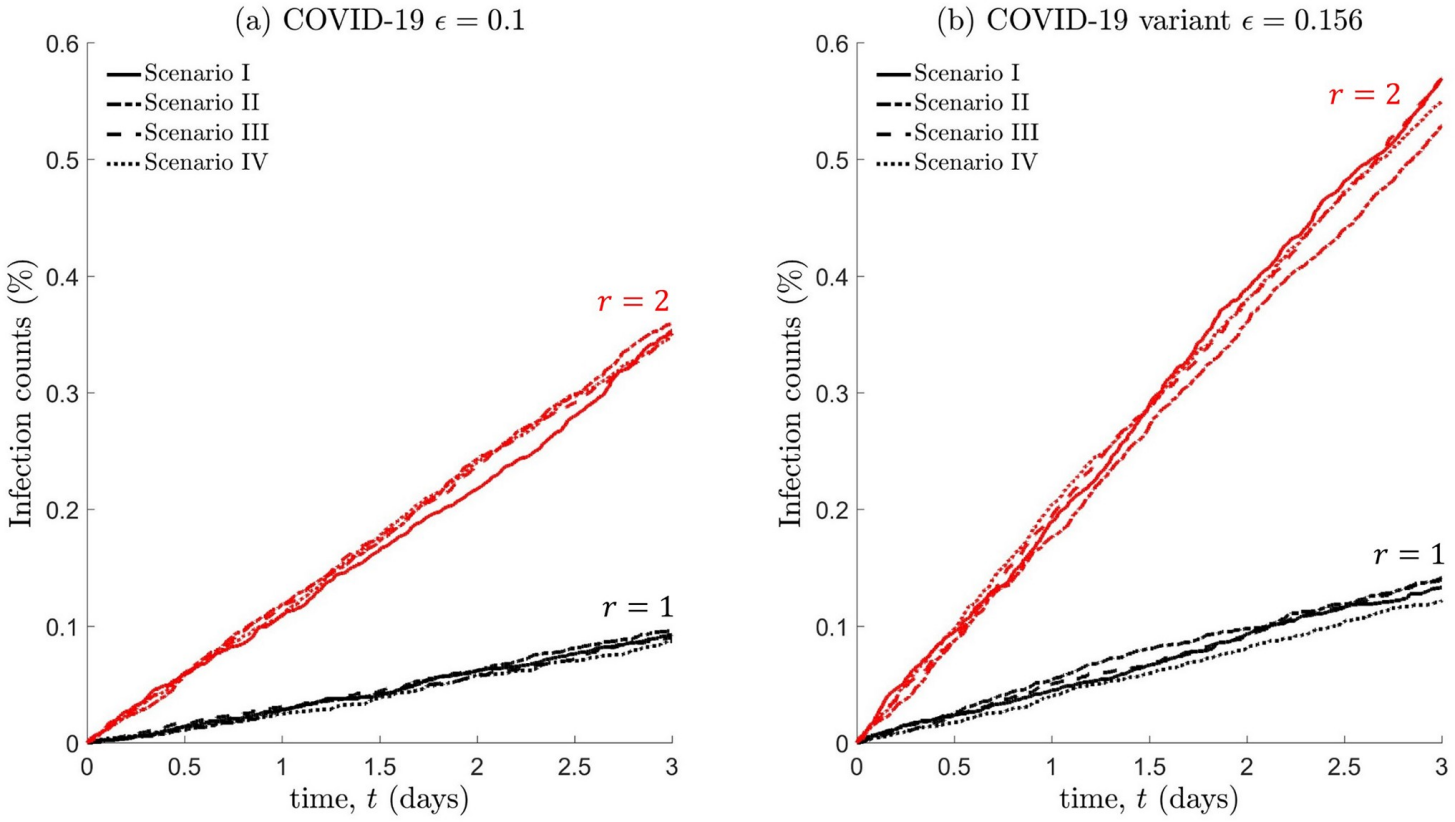
Infection counts for a homogeneously distributed population, under the lockdown scenarios I-IV shown in Fig 4. Each individual in the population performs a SRW. The interaction radius is *r* = 1m (black) or *r* = 2m (red), with transmission probabilities (a) *ϵ* = 0.1 (COVID-19) or (b) *ϵ* = 0.156 (variant).

As expected, Fig 8 shows that infection counts increase with a larger interaction radius, however, the differences in these counts can be further exacerbated with an increase in virus transmissibility. On comparing infection counts in case of free movement (no lockdown, scenario I) and restricted movement within confined space (lockdown scenarios II-IV), we find that the impact is negligible, at least in the short-term. This appears to be a direct consequence of homogeneity in the population spatial distribution.

**Fig 9:**
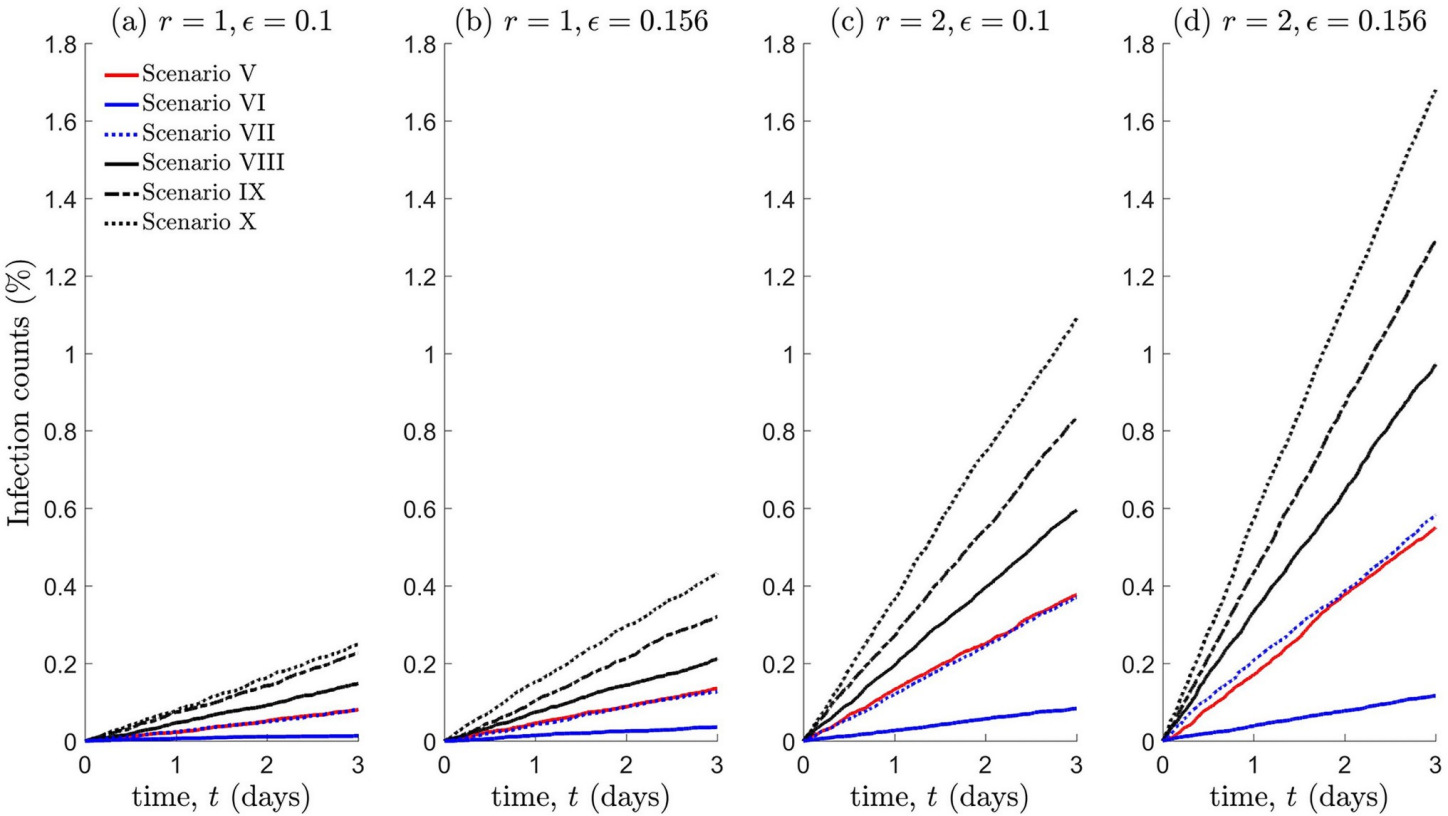
Infection counts for a heterogeneously distributed population, under the lockdown scenarios V-X shown in Fig 6. All other details are the same as that in the caption of Fig 8.

Fig 9 shows that infection counts vary significantly if the population is distributed unevenly, and heavily depends on the spatial distribution of infectious individuals prior to the lockdown. On comparing scenarios V (no lockdown) and VII, we find that lockdowns are ineffective, i.e., if a small proportion of infectious individuals are present within the overcrowded region. Scenarios VIII-X demonstrate that lockdowns can produce adverse effects if the proportion of infectious individuals exceeds a certain threshold, with a substantial increase in infection levels due to more frequent contacts. This negative impact of lockdowns is more realised with a larger interaction radius and if the virus is more transmissible. Scenario VI indicates that lockdowns should be enforced if infectious individuals are predominately located in sparsely populated regions, as a pre-emptive measure to deter population mixing with high density regions.

## Discussion

Traditional models of infectious diseases usually assume that susceptible and host populations mix readily, and fail to capture the interactions between individuals [82], whereas mechanistic models account for the spatial proximity between individuals and explicitly model the contact process—which is directly related to the disease transmission process [11, 21]. Also, much focus in the literature is on the long-term effects of epidemic spread, which is important to address questions related to disease spread/control and the resulting socio-economic impacts etc. [83]. In contrast, very few studies focus on the short-time scale of the infection dynamics, despite growing attention towards asymptomatic yet infectious carriers, as they are hard to track and could be a critical factor in the spread of some diseases [84]. In this paper we set out to investigate how (cumulative) infection counts are driven by the interactions between susceptible and infectious individuals on a short-time scale, whilst considering different types of movement behaviours. An individual mechanistic modelling approach was used based on RWs, which is increasingly recognised as a fundamental tool of infectious disease epidemiology [11, 21]. In addition, we analysed how the spatial distribution of the population can impact the effectiveness of lockdowns.

We found that cumulative infection levels did not vary much with an increase in forward persistence, and also remained constant in the case of faster population dispersal (suggesting that, on average, the same number of contacts over a fixed time period). This means that, on a short-time scale (∼3 days), the movement pattern of a population has no effect, and thus cannot be used to explain the infection dynamics. In practice, lockdowns can be enforced at national or at local levels, and aim to limit interactions between people through a variety of methods e.g., stay at home orders, banning travel to or from an area at a very high alert level etc. In this study, we focused on a specific type of lockdown, that enforce individuals to be confined within a specific region. We found that the effectiveness of lockdowns depends significantly on how the population (including infectious individuals) are distributed over space. In the case of a homogeneous population, we found that the increase in infection levels was approximately the same, irrespective of different lockdown scenarios, where other parameters played a more important role, such as the interaction radius, and virus transmissibility. This can be explained by the average number of contacts is expected to be approximately the same over the whole region, despite being greater over a sub-region where more infectious carriers are present. In the case of a heterogeneous population, we find that lockdowns are effective if a large proportion of infected individuals are distributed in sparsely populated sub-regions to reduce contacts by limiting population mixing. However, lockdowns can be ineffective if a small proportion of carriers are located within high density areas, and can also lead to an increase in infection levels if a considerable large proportion of carriers are present. This demonstrates that lockdown strategies should not only be based on rising levels of infection, but should also account for the distribution of infectious carriers and local population densities. The simulation scenarios can be used to better understand the first generation of disease transmission on a short-time scale, whether it be originated at the Huanan Seafood Market in Wuhan, China, or an unknown source of the infection [20]. More generally, the results can help to develop prevention policies once a newly infected individual is identified in an area where previously the disease was absent.

One important aspect of this study is that no further disease transmission was assumed once individuals in the susceptible population were infected, which is reasonable on a short-time scale as there is an estimated time lag of approximately 3 days before an infected individual becomes infectious. To investigate the long-term effects, the simulation model would need to be refined to account for further transmission. Under such a scenario, one could expect richer infection dynamics where individual movement plays an important role, since contacts between individuals is partly driven by the distribution of the population over space. Also, one could estimate variations in a key epidemiological metric—the effective reproduction number (*R*), i.e., the average number of secondary cases per infectious case in a population [85]. Moreover, the interplay between host movements and various lockdown scenarios on this time scale warrants further investigation. On a longer-time scale, lockdowns are expected to be effective [80, 86], and there are recommendations that they should remain in place for a time period of about 60 days [87]—however, it is still unclear when and how a lockdown should be enforced [88]. It is plausible to hypothesize that the exact timing may depend on the movement patterns of individuals and the resulting population distribution. Such information is vital for stakeholders (government, health officials, policy makers etc.), as some countries are past their (first or even second) epidemic peak [89, 90], and a third wave of the pandemic is anticipated.

The simulations considered in this study modelled those individuals who were in close spatial proximity of the infectious host as instantaneously infected, albeit with different contact radii and transmission probabilities. This allows disease modellers to identify and quantify ‘near misses’ and to explore possible alternative epidemic outcomes given shifts in these parameters [11]. By extension, one could consider a continuous-time RW model for the movement of individuals in the population, which allows for random waiting times between steps [91, 92]. Under such a description, the susceptible host is infected not only if it is in close contact with the infectious host, but also requires that the waiting time exceeds a certain threshold. An improvement to the model would consider the transmission probability to be directly related to the duration of the interaction time-period. A basic assumption of the discrete time RW model is that each walker moves independently of each other, however, in some context-specific scenarios, individuals may purposely avoid crowded areas during a pandemic—and therefore the movement can be dependent on the local density. A future research direction would be to utilize or develop existing models on human crowd dynamics [93, 94] or animal collective movement (which is well studied in the discipline of movement ecology [95, 96]) towards the study of infectious diseases. Moreover, if contact rates and transmission probabilities can be estimated from epidemic/movement data, mechanistic models could prove to provide a powerful modelling framework for a broader category of diseases [11].

### List of abbreviations

World Health Organization (WHO), Centers for Disease Control and Prevention (CDC), Random Walk (RW), Simple Random Walk (SRW), Correlated Random Walk (CRW), Lévy Walk (LW), Correlated Lévy Walk (CLW).

## Supporting information

S1 File (PDF)
