# Supplementary material for: Mechanistic modelling of COVID-19 and the impact of lockdowns on a short-time scale
Source: PLoS One. 2021 Oct 18;16(10):e0258084. doi: 10.1371/journal.pone.0258084 (PMC8523076; doi:10.1371/journal.pone.0258084)
Supplement: S1 File — (PDF) [file pone.0258084.s001.pdf]

Supporting information

Equivalence between RWs

Two isotropic RWs where at least one of them has an infinite MSD can be compared by relating the distributions of step lengths $\lambda_{\mathrm{A}}(l;\sigma)$ and $\lambda_{\mathrm{B}}(l;\gamma)$, where σ, γ are the respective movement parameters. If we consider these distributions to have the same characteristic scale length L and survival probability p defined as:

$$\mathbb{P}(l > L) = p, \tag{S1}$$

one gets:

$$\int_L^\infty \lambda_{\mathrm{A}}(l;\sigma)dl = \int_L^\infty \lambda_{\mathrm{B}}(l;\gamma)dl = p, \tag{S2}$$

with ratio of distribution parameters:

$$\frac{\gamma}{\sigma} = \xi(p) \tag{S3}$$

expressed solely as a function of the survival probability p. However, these conditions can be satisfied for an arbitrary value of p. To determine p uniquely an additional constraint is required, and therefore we consider the (squared) $\mathbb{L}_2$ norm, defined as:

$$\mathbb{L}_2^2 = \int_0^\infty \left[\lambda_{\mathrm{B}}(l;\gamma) - \lambda_{\mathrm{A}}(l;\sigma)\right]^2 dl. \tag{S4}$$

From Eq (S3) this can be written as:

$$\mathbb{L}_2^2 = \int_0^\infty \left[\lambda_{\mathrm{B}}(l;\sigma\xi(p)) - \lambda_{\mathrm{A}}(l;\sigma)\right]^2 dl, \tag{S5}$$

which is now expressed only in terms of p and σ. The optimal survival probability, say $p = p^*$, which minimizes Eq (S5), can be found by solving:

$$\left.\frac{d}{dp}\mathbb{L}_2^2(p;\sigma)\right|_{p=p^*} = 0. \tag{S6}$$

Note that minimizing the squared norm is the same as minimizing the $\mathbb{L}_2$ norm. Once p^* is computed, the optimal characteristic scale length L^* can be determined from Eq (S2), and the scale parameters can be related from Eq (S3) as $\gamma = \xi^* \sigma$ with optimal ratio $\xi^* = \xi\left(p^*\right)$. This method can also be applied for correlated RWs with the same distribution of turning angles.

Consider now the example where we seek to compare a SRW with Rayleigh distributed step lengths:

$$\lambda_{\mathrm{A}}(l) = \frac{l}{\sigma^2} \exp\left(-\frac{l^2}{2\sigma^2}\right), \tag{S7}$$

and a LW with folded-Cauchy distributed step lengths:

$$\lambda_{\mathrm{B}}(l;\gamma) = \frac{2\gamma}{\pi(\gamma^2 + l^2)}. \tag{S8}$$

From the equivalence condition in Eq (S2), one gets:

$$L = \sigma\sqrt{-2\ln p} = \gamma \tan\left[\frac{\pi}{2}(1-p)\right], \tag{S9}$$

and on rearranging, we have that:

$$\xi(p) = \frac{\gamma}{\sigma} = \sqrt{-2\ln p}\cot\left[\frac{\pi}{2}(1-p)\right]. \tag{S10}$$

Following this, the squared norm in Eq (S5) can be evaluated analytically to obtain:

$$\mathbb{L}_2^2 = \frac{1}{\pi\sigma}\cdot\left(\frac{1}{\xi} - 2\xi\exp\left(\frac{\xi^2}{2}\right)\mathrm{E}_1\left(\frac{\xi^2}{2}\right) + \frac{\pi\sqrt{\pi}}{4}\right), \tag{S11}$$

where $\mathrm{E}_1(\tau) = \int_\tau^\infty \frac{1}{\tau'}\exp(\tau')d\tau'$ is a form of the exponential integral. The derivative of the above Eq (S11) can be written as:

$$\frac{d\mathbb{L}_2}{dp} = -\frac{1}{2\pi\sigma\mathbb{L}_2}\psi(p)s'(p), \tag{S12}$$

with the optimality function ψ given as:

$$\psi(p) = \frac{1-4\xi^2}{\xi^2} + 2\left(1+\xi^2\right)\exp\left(\frac{\xi^2}{2}\right)\mathrm{E}_1\left(\frac{\xi^2}{2}\right) \tag{S13}$$

whose zero occurs at $p^* = 0.658$ corresponding to the optimal distribution parameter ratio:

$$\xi^* = \frac{\gamma}{\sigma} = 1.536. \tag{S14}$$
